# Aerodynamic efficiency of a bioinspired flapping wing rotor at low Reynolds number

**DOI:** 10.1098/rsos.171307

**Published:** 2018-03-14

**Authors:** H. Li, S. Guo

**Affiliations:** Centre of Aeronautics, Aerospace, Cranfield University, Cranfield, Beds, UK

**Keywords:** aerodynamic efficiency, flapping wing rotor, passive rotation, bioinspiration, micro air vehicle

## Abstract

This study investigates the aerodynamic efficiency of a bioinspired flapping wing rotor kinematics which combines an active vertical flapping motion and a passive horizontal rotation induced by aerodynamic thrust. The aerodynamic efficiencies for producing both vertical lift and horizontal thrust of the wing are obtained using a quasi-steady aerodynamic model and two-dimensional (2D) CFD analysis at Reynolds number of 2500. The calculated efficiency data show that both efficiencies (propulsive efficiency-*η*_p_, and efficiency for producing lift-*P_f_*) of the wing are optimized at Strouhal number (*St*) between 0.1 and 0.5 for a range of wing pitch angles (upstroke angle of attack *α*_u_ less than 45°); the *St* for high *P_f_* (*St* = 0.1 ∼ 0.3) is generally lower than for high *η*_p_ (*St* = 0.2 ∼ 0.5), while the *St* for equilibrium rotation states lies between the two. Further systematic calculations show that the natural equilibrium of the passive rotating wing automatically converges to high-efficiency states: above 85% of maximum *P_f_* can be obtained for a wide range of prescribed wing kinematics. This study provides insight into the aerodynamic efficiency of biological flyers in cruising flight, as well as practical applications for micro air vehicle design.

## 1. Introduction

Flapping wing based propulsion has several aerodynamic benefits for the application of micro air vehicles (MAVs), as demonstrated by the superior flight skills of natural flyers such as insects or birds. In particular, flapping wing produces higher lift force than conventional aerofoil at an angle of attack (AoA) above the stall angle, due to the delayed stall of the leading edge vortex (LEV) [1–3]. On the other hand, flying insects and birds have shown the extraordinary capability of vertical take-off, landing, hovering and manoeuvrability. These features of the flapping wing have brought a strong interest in developing MAVs that mimic the wing motion of insects or birds [4,5].

For aeronautical vehicles and flying animals, the wings produce significant lift to support weight in the air, the aerodynamic efficiency is often defined by the cost of energy to stay aloft or to travel a certain distance, which is associated with lift production. For example, the current studies on insect flight primarily focus on a ‘normal hovering’ state, where the aerodynamic force of the flapping wings averages to a net lift primarily to support the weight in the air. The aerodynamic efficiency of the wing is therefore mainly concerned with the production of lift for a given power. On the other hand, moving animals such as swimming fish and cruising flyers use their wings or fins to produce thrust against the drag of the fluid, for which the propulsive efficiency associated with thrust production is often used to measure the efficiency of their movement of wings or fins. For flying and swimming animals, a dimensionless parameter which describes the kinematics of their wings or fins is the Strouhal number *St* [6–8]. This dimensionless number is known to govern a well-defined structure of the wake shed from an oscillating aerofoil in the free stream, and is closely related to the efficiency of flying and swimming animals for propulsion [6,8]. In particular, optimum propulsive efficiency is found for wing motions with *St* lying between 0.2 and 0.4, corresponding to a stably formed wake structure and average velocity profile equivalent to a jet [6–10]. Further extensive literature reviews on data from flying and swimming animals showed that many animals cruise at this interval of *St* [6,8].

The existing studies on flapping wing efficiencies have been primarily focused on one of the above two aspects. For the normal hovering kinematics of insects, because the body assumes no forward speed, the flapping wing produces no net thrust in a flapping cycle. Thus, the aerodynamic efficiency for producing lift is of primary concern for such wing motions. On the other hand, most study on the undulatory swimming fish tails have been devoted to propulsion, while the force perpendicular to the free stream in the form of lift is of secondary factor. However, natural flyers in cruising flight use their flapping wings to generate both lift and thrust. The aerodynamic efficiency of such wing motion in terms of both propulsive efficiency and efficiency of lift production has received less study.

**Figure 1.**
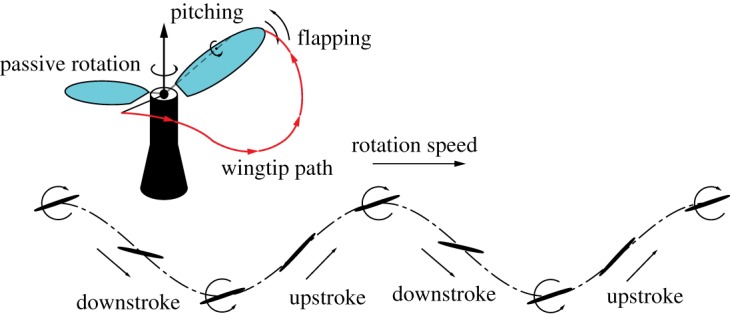
Flapping and passive rotation kinematics of FWR.

One particularly interesting kinematics is a wing that flaps and rotates at the same time. Vandenberghe *et al*. [11] studied these kinematics experimentally with a rectangular wing mounted on a vertical shaft and free to rotate horizontally. As the wing flaps up and down above a threshold frequency, it starts to rotate and finally reaches a stable speed with *St *∼ 0.26 for a range of input frequencies. Similar wing kinematics has been proposed for the design of new helicopters without the reaction torque [12]. Guo *et al*. [13] investigated a new type of bioinspired flapping wing vehicle, namely the flapping wing rotor (FWR), for which a pair of antisymmetric configured wings is rotated by the aerodynamic thrust produced by vertical flapping motion, as illustrated in figure 1. The positive average aerodynamic lift can be obtained by varying the wing pitch angle in an asymmetric manner. Further numerical investigation has shown that the flow on the flapping and rotating wing forms compactly attached three-dimensional (3D) vortex ring structure which connects the LEV, trailing edge vortex and wingtip vortex that enhances lift production [14]. Li *et al*. [15] and Wu *et al*. [16] used quasi-steady (QS) aerodynamic analysis and CFD models, respectively, and showed that the FWR kinematics in hovering flight can produce higher lift coefficient than the conventional insect-like flapping wings and rotary wing. The aerodynamic efficiency of the FWR for lift production (defined by the dimensionless power factor *P_f_* [17,18]) is standing between the other two conventional types.

For animals in cruising flight, the equilibrium between the thrust produced by the flapping wing and the aerodynamic drag results in a wing kinematics pattern that closely resembles the wing motion of the FWR. In this study, investigations have been made into the aerodynamic efficiency for producing both vertical lift and horizontal thrust of the FWR kinematics. A 3D QS aerodynamic model which adapts the empirical coefficients obtained from high-fidelity CFD simulation is used to calculate the aerodynamic force and power of the wing. Additionally, analysis using a two-dimensional (2D) CFD model is carried out to capture the transient status of the flow field and unsteady forces. A typical wing model of elliptical shape and aspect ratio λ = 3.6 and semi-span *R* = 0.098 m is chosen for the FWR model. The wing flapping motion follows a simple harmonic function (SHF) at a constant rotating speed. The wing flapping frequency is *f* = 10 Hz, flapping amplitude *Φ* = 70° corresponding to a Reynolds number of 2500.

The results of this study show that both the propulsive efficiency and efficiency for producing lift of the FWR wing peaks within a narrow interval of *St*: between 0.1 and 0.5 for certain range of wing pitch angles, which agrees closely with reported data of natural flyers in cruising flight [8]; however, the *St* for high efficiency of lift and high efficiency of propulsion in general differs. The higher propulsive efficiency corresponds to *St* between 0.2 and 0.5 and the higher efficiency of lift corresponds to *St* between 0.1 and 0.3; in particular, the *St* for the rotational equilibrium state of the wing lies between the maximum propulsive efficiency state and the maximum efficiency of lift state. Furthermore, systematic calculations show that high efficiency of lift (above 85% with respect to maximum *P_f_*) can be obtained at the natural equilibrium state of the wing for wide range of prescribed wing kinematics. Insights of the results for biological flyers in cruising flight as well as for MAVs design are provided.

## 2. Model and method

### 2.1. Wing kinematics definition

**Figure 2.**
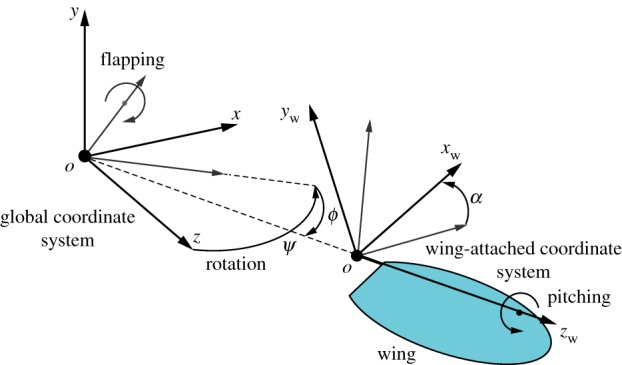
Coordinate systems definition for the FWR wing.

The coordinate system to define the FWR wing motion is shown in figure 2. The kinematics of the wing is defined by three elementary motions: rotation, flapping and pitching. The wing rotates about the vertical *y*-axis, and the rotation speed is fixed constant and indicated by $\dot{\psi }$. The dimensionless wing rotation speed is defined as

2.1 $$\eta =\frac{\dot{\psi }}{2\Phi f},$$

where Φ is the flapping angle amplitude and *f* is the flapping frequency.

The wing flaps vertically with the flapping angular velocity $\dot{\phi }$ described by the SHF:

2.2 $$\dot{\phi }=-\pi f\Phi \;\sin(2\pi \tau ),$$

where *τ* = *ft* is the dimensionless time ranging from 0 ∼ 1 in a flapping period.

The wing pitches at the same frequency *f*, with a phase shift of *π*/2. The pitch motion of the wing is confined to stroke reversals. At the mid-upstroke and mid-downstroke, the wing has angle of attack (AoA) denoted by *α*_u_ and *α*_d_, respectively. In the reversal phase at the end of each stroke, the pitch angular velocity of the wing $\dot{\alpha }$ is described by the following equation:

2.3 $$\dot{\alpha }=\frac{2f(\alpha_{u}-\alpha_{d})}{\Delta \tau_{r}}\left\{{\left(-1\right)}^{\left[2\tau +0.5\right]}-\text{cos}\left(\frac{4\pi \tau }{\Delta \tau_{r}}-[2\tau -0.5]\pi \right)\right\}\;,$$

where $\Delta \tau_{r}=0∼1$ indicates the dimensionless wing pitch time with respect to the flapping period, and the bracket notation [·] indicates the floor function giving the greatest bounding integer. In this study, the wing pitch time takes half of the flapping period, corresponding to $\Delta \tau_{r}=0.5$.

### 2.2. Quasi-steady model and dimensionless parameters

**Figure 3.**
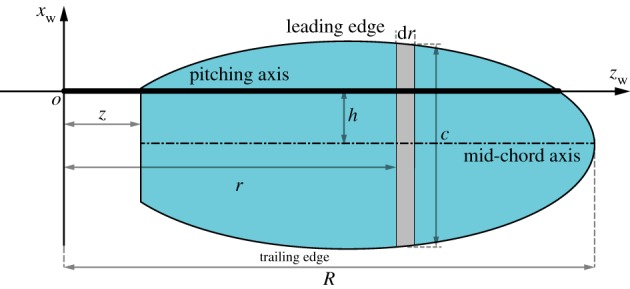
Geometry and parametric definitions of the wing.

The wing planform is in ellipse as illustrated in figure 3. The pitching axis of the wing (*z*_w_) is located near the leading edge; *c* is the local chord length at a 2D wing strip d*r*; *h* is the vertical distance between the mid-chord axis and the pitching axis of the wing. The pitching axis of the wing is taken to be located at 0.25 chord [19,20], corresponding to *h* = 0.25 *c*.

The aerodynamic force of the wing is solved by employing a QS aerodynamic model. The details of the model are provided in the previous study [15]. On a 2D wing strip, the aerodynamic forces and pitch moment in the local wing-attached frame (*x*_w_, *y*_w_ and *z*_w_, as shown in figure 2) are obtained by the equations:

2.4 $$\begin{array}{rl} dF_{x} & =\left\{\frac{1}{2}\rho U^{2}C_{H}c+[\lambda_{y}u_{y}\omega_{z}+\lambda_{y\omega }\omega_{z}^{2}]\right\}\text{d}r, \end{array}$$

2.5 $$\begin{array}{rl} \text{d}F_{y} & =\left\{\frac{1}{2}\rho U^{2}C_{V}c+C_{\mathrm{ROT}}\rho U\omega_{z}c^{2}-[\lambda_{y}{\dot{u}}_{y}+\lambda_{y\omega }{\dot{\omega }}_{z}]\right\}\text{d}r \end{array}$$

2.6 $$\begin{array}{rl} \;\text{and}\;\;\text{d}\tau_{z} & =\left\{-\frac{1}{2}\rho U^{2}C_{V}{\hat{x}}_{\mathrm{CP}}c^{2}-\frac{1}{2}\rho \omega_{z}|\omega_{z}|C_{\mathrm{RD}}{\hat{x}}_{\mathrm{RD}}c^{4}+[\lambda_{y}u_{x}u_{y}+\lambda_{y\omega }({\dot{u}}_{y}+u_{x}\omega_{z})+\lambda_{\omega }{\dot{\omega }}_{z}]\right\}\text{d}r, \end{array}$$

where *ρ* is the air density; *U* is the translational velocity; *ω_z_* and ${\dot{\omega }}_{z}$ are the wing pitch rate and pitch acceleration; *u_x_*, *u_y_* and ${\dot{u}}_{y}$ are the translational velocity components in the *x*_w_- and *y*_w_-axes and the acceleration; λ*_y_* and λ*_yω_* are the added mass force coefficients, which are given by $\lambda_{y}=\pi \text{/}4\rho c^{2}$ and $\lambda_{y\omega }=\pi \text{/}4\rho hc^{2}$; λ*_ω_* is added mass moment coefficient, which is given as: $\lambda_{\omega }=\pi \text{/}4\rho h^{2}c^{2}+\pi \text{/}128\rho c^{4}$ [21].

The QS aerodynamic coefficients *C_H_*, *C_V_* and *C*_ROT_ are empirical coefficients for the translational force and rotational force [20,22]; *C*_RD_ is the rotational damping moment coefficient; ${\hat{x}}_{\mathrm{CP}}$ is the dimensionless centre of pressure (CP) of the translational force; ${\hat{x}}_{\mathrm{RD}}$ is the dimensionless location of the rotational damping force; The relation of the empirical coefficients with the effective AoA *α_e_* are provided in our previous study [15].

The 2D aerodynamic forces and pitch moment for each wing strip are integrated along the wing-span to obtain the 3D forces and moments. The vertical lift force and rotational moment of the wing can then be obtained by projecting the 3D force and moment vectors onto the global *y*-axis of the coordinate, as shown in figure 2. The lift and rotational moment coefficients are defined as

2.7 $$C_{l}=\frac{l}{0.5\rho U_{2}^{2}S}\;$$

and

2.8 $$C_{m}=\frac{m}{0.5\rho U_{2}^{2}S\bar{c}},$$

where *l* is the vertical lift force and *m* is the rotational moment, i.e. moment along the global *y*-axis, $\bar{c}$ is the mean chord length, *S* is the wing area. The reference velocity *U*_2_ is defined by

2.9 $$U_{2}=2\Phi fr_{2},$$

where $r_{2}=\sqrt{\int r^{2}\text{d}S\text{/}S}$ is the radius of the second moment of wing area. The mean coefficients$\;{\bar{C}}_{l}$ and ${\bar{C}}_{m}$ are defined similarly with the mean lift force $\left(\bar{l}=\int_{0}^{T}l\text{d}t\text{/}T\right)$ and rotational moment $\left(\bar{m}=\int_{0}^{T}m\text{d}t\text{/}T\right)$ put into the equations instead of the instantaneous values.

The dimensionless parameters that govern the flow and the shedding of vortices are the Strouhal number and the reduced frequency. The Strouhal number *St* is defined by

2.10 $$St=\frac{fA}{U},$$

where the characteristic width *A* is taken to be the stroke amplitude at the wingtip, and the forward speed *U* is taken to be the rotation speed of the wing at the wingtip [8]. For a 2D aerofoil, the reduced frequency *k*_c_ is defined by

2.11 $$k_{c}=\frac{2\pi fc}{U_{c}},$$

where *U*_c_ is the rotation speed at the specific chord.

### 2.3. Aerodynamic power and efficiency measures

The mean aerodynamic power over a flapping cycle *T* can be obtained by summing the aerodynamic power of each independent axis of rotation:

2.12 $$\bar{P}=\underset{i=x,y,z}{\sum }{\bar{P}}_{i},$$

where ${\bar{P}}_{i}=-\int_{0}^{T}\omega_{i}\tau_{i}\text{d}t\text{/}T$ is the mean aerodynamic power of the *i*th axis. The mean aerodynamic power coefficient can be defined as

2.13 $${\bar{C}}_{P}=\frac{\bar{P}}{0.5\rho U_{2}^{3}S}.$$

The mean aerodynamic power $\bar{P}$ is the total power required for the wing to overcome the fluid forces. Therefore, when measuring the efficiency of lift production, ${\bar{C}}_{P}$ can be used directly to define the dimensionless power factor *P_f_* [17,18], which measures the power efficiency of flying animals and vehicles for sustaining a specific weight:

2.14 $$P_{f}=\frac{{\bar{C}}_{l}^{1.5}}{{\bar{C}}_{P}}.$$

However, when measuring the propulsive efficiency, the mean aerodynamic power of each independent axis ${\bar{P}}_{i}$ needs to be treated differently. The propulsive efficiency of the wing *η*_p_ is defined by the ratio of aerodynamic power output (for propulsion) to the power input:

2.15 $$\eta_{p}=\frac{\left|{\bar{P}}_{y}\right|}{\bar{P}-{\bar{P}}_{y}}.$$

When the flapping wing is producing positive propelling moment, this definition agrees with the usual definition of propulsive efficiency for oscillating foils in the free stream [6,7].

## 3. Comparison of 3D quasi-steady and 2D unsteady forces

This study employs a 3D QS aerodynamic model for modelling the forces and power of the FWR wing. Additionally, a 2D CFD analysis is carried out using the commercial CFD solver ANSYS Fluent, which solves the 2D unsteady, incompressible Navier–Stokes equations based on a finite volume method. The accuracy of this solver has been extensively validated against several experimental and numerical studies in flapping wing aerodynamics [23]. The 2D aerofoil is chosen as flat plate of 2% thickness. The choice of the simplified models is due to the efficiency for computing the various wing kinematic cases.

In this investigation, the QS model and 2D CFD model are compared with the high-fidelity 3D CFD results from the previous study. A particular kinematic case from Wu *et al.* [16] is chosen with the kinematic parameters of the wing specified by: Φ = 70°, *α*_u_ = 60°, *α*_d_ = −20° and the rotation speed *η* = 1.10. In this case, the wing model is of rectangular shape and with aspect ratio 5.8 $\left(\lambda =R\text{/}\bar{c}\right)$. The wing semi-span *R* and the flapping frequency *f* are given by *R* = 0.098 m and *f* = 10 Hz, corresponding to *Re* ∼ 1600 ($Re=U_{2}\bar{c}\text{/}\upsilon$, where *U*_2_ is the mean flapping velocity at the radius of the second moment of wing area *r*_2_, and $\bar{c}$ is mean chord length). For the 2D CFD analysis, a series of wing chords located along the wing-span (ranging between 0.2 and 0.7 wing-spans) is taken for investigation. To compare the 2D model with 3D results, the thrust coefficient of the 2D results ($C_{T}=T\text{/}0.5\rho {\bar{U}}^{2}c$, where *T* is the thrust force and $\bar{U}$ is the local mean flapping velocity) is converted to the 3D rotational moment coefficient *C*_m_ using a scale factor of λ(*I*_3_/*I*_2_) obtained from a standard blade element analysis, λ is the wing aspect ratio and *I_k_* is the *k*th dimensionless moment of wing area defined by the equation:

3.1 $$I_{k}=\frac{\int r^{k}\text{d}S}{R^{k}S}.$$

**Figure 4.**
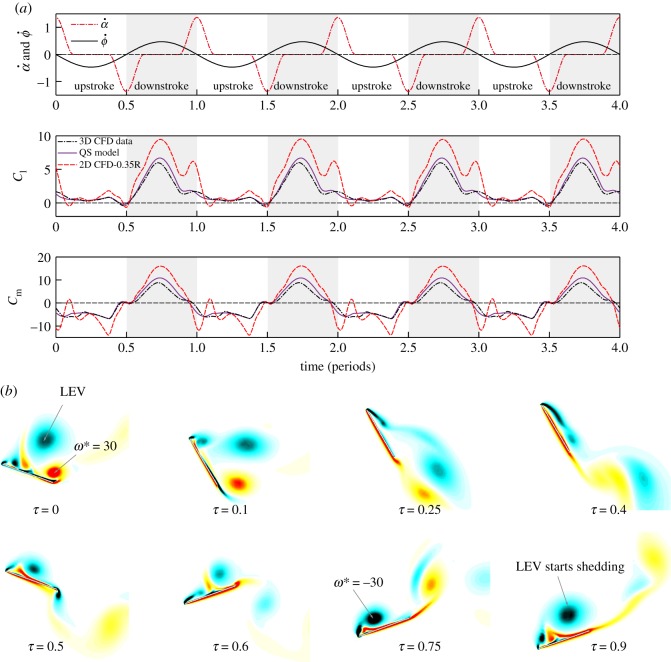
(*a*) Comparison of *C*_l_ and *C*_m_ by QS, 2D and 3D CFD model results; (*b*) Contour of flow vorticity for 0.35R (*k*_c_ = 1.15) 2D wing (red and black colour indicates anti-clockwise and clockwise rotating vortices, respectively).

The time courses of the forces and rotational moments by different models are shown in figure 4*a*, and the flow field for the 2D wing chord is shown in figure 4*b*. All the cases have the same *St* = 0.45; the 2D CFD result presented here is taken at 0.35 wing-span, with reduced frequency *k*_c_ = 1.15. The full spectrum of 2D results at different span-wise locations ranging between 0.2R and 0.7R is provided in the electronic supplementary material.

From the results in figure 4*a*, it is clear that the time courses of the 3D QS forces and moments agree very well with the 3D CFD results (a more comprehensive validation of the QS model for both transient and mean aerodynamic forces is provided in [15]); while for the given wing chord at 0.35 wing-span, the 2D CFD results appear larger due to the well-known downwash of the wingtip vortex for a 3D wing. However, the variations of the 2D transient forces agree qualitatively with the 3D transient forces.

In figure 4*b*, the flow over the 2D wing chord shows a dynamic formation and shedding of vortices that closely resembles the dynamic stall of conventional aerofoil. In the downstroke, a strong LEV first forms on the upper surface until a reverse flow emerges from the trailing edge and the LEV starts to shed from the surface. The shedding of the LEV is near the end of the downstroke, corresponding to a decrease in lift coefficient.

The particular choice of wing chord at 0.35R to represent the flow field follows from the observation that the shedding of LEV is at the end of the downstroke. In previous studies on 2D flapping wing, the frequency of LEV shedding was shown to play a significant role in determining the transient forces. Lewin & Haj-Hariri [24] and Wang [25] used numerical simulation to analyse an aerofoil in 2D flow undergoing heaving oscillating. They show that the dimensionless reduced frequency *k*_c_ and the Strouhal number *St* serve to govern the time scale associated with the growth and shedding of the vortices on the wing. The *k*_c_ is the primary factor governing the LEV shedding and *St* the secondary factor related to the growth of LEV. For a smaller value of *k*_c_, the LEV tends to separate and advect along the freestream, leading to an early separation of the LEV; while for larger *k*_c_, the LEV tends to separate later and stays longer on the wing in each flapping stroke. Similar flow phenomenon is observed in this study. The LEV on the outer wing chords (with smaller *k*_c_) tends to separate early while it stays longer on the inner wing chords with larger *k*_c_ (see the electronic supplementary material).

In this study, in order to obtain the qualitative characteristics of the flow, the 2D CFD analysis is taken at different span-wise locations of the FWR wing, which has the same *St* but different *k*_c_ (ranging between 0.6 and 2.0 for different *St* cases). The particular cases of the 2D results with LEV shedding frequency that matches with the flapping frequency of the wing (i.e. LEV shed at the end of the downstroke) is then chosen to represent the flow field, the resulting transient forces of the 2D calculations are found in qualitative agreement with 3D models.

## 4. Results and discussion

### 4.1. Propulsive efficiency versus the efficiency of lift

The FWR kinematics makes use of aerodynamic thrust produced by flapping motion to drive the wings to rotate about the vertical axis. At the same time, lift is obtained by biasing the pitch angle of the wing in the upstroke and downstroke. In this study, both the propulsive efficiency *η*_p_ and the efficiency for producing lift *P_f_* are investigated for the FWR kinematics. These two efficiency measures are calculated in different kinematic conditions defined by the dimensionless *St* and wing pitch angles.

**Figure 5.**
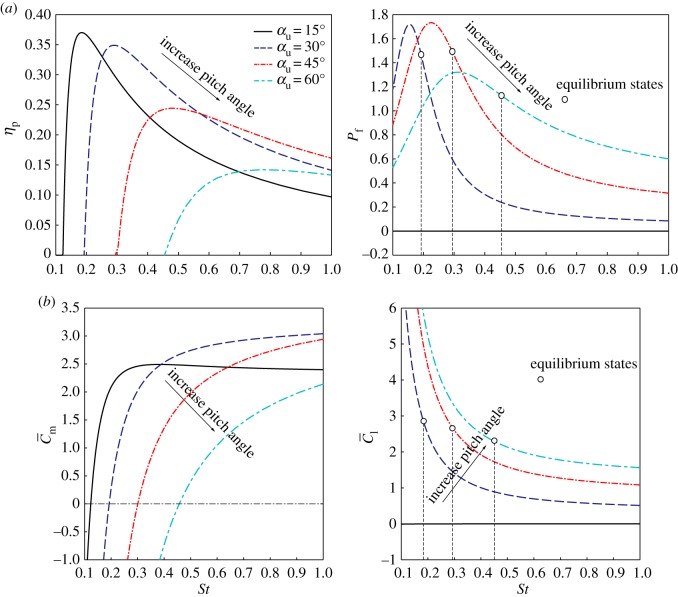
(*a*) Propulsive efficiency *η*_p_ and efficiency for producing lift *P_f_* against the *St*; (*b*) mean rotational moment and lift coefficients ${\bar{C}}_{m}$ and ${\bar{C}}_{l}$ against the *St*. All cases have *α*_d_ *=* −15°; open circles indicate rotational equilibrium states where ${\bar{C}}_{m}=0$.

An FWR wing model with wing shape illustrated in figure 3, wing semi-span *R* = 0.098 m, aspect ratio λ = 3.6, flapping amplitude Φ = 70° and flapping frequency *f* = 10 Hz at *Re* ∼ 2500 is taken for this study. The wing pitch angles are defined for four cases, varying from symmetric pitching to asymmetric pitching as: *α*_d_ = −15° and *α*_u_ = 15°, 30°, 45°, 60°. The *S_t_* for each of the above cases are chosen to vary between S*t* = 0.1 ∼ 1, which determines the rotation speeds of the wing uniquely (*η *= 0.5 ∼ 5 for the given *St* range). The computed results for aerodynamic efficiencies (*η*_p_ and *P_f_*) and force (moment) coefficients (${\bar{C}}_{l}$ and ${\bar{C}}_{m}$) against the *St* are shown in figure 5. The *St* for maximum *η*_p_ and *P_f_* at different *α*_u_ cases is given in table 1.

**Table 1.** The *St* for maximum *η*_p_ and *P_f_* for fixed *α*_d_ = −15°.

kinematic case	maximum *η*_p_	*St* for maximum *η*_p_	maximum *P_f_*	*St* for maximum *P_f_*
*α*_u_ = 15°	0.37	0.19	0	/
*α*_u_ = 30°	0.35	0.29	1.72	0.15
*α*_u_ = 45°	0.24	0.48	1.73	0.22
*α*_u_ = 60°	0.14	0.77	1.32	0.31

As shown in figure 5*a*, the variation of the propulsive efficiency *η*_p_ and the efficiency of lift *P_f_* against the *St* follows a similar trend. As the *St* increases from 0.1 to 1, both *η*_p_ and *P_f_* first increase rapidly to the maximum values and then decrease. It is also observed that both the maximum *η*_p_ and *P_f_* occur within a narrow interval of *St* = 0.15 ∼ 0.5 when the wing upstroke pitch angle is within 15° ∼ 45°. However, these two efficiencies appear to be complementary with respect to each other, hence do not reach maximum at the same time. In one of the extreme cases at the lower end of *St* = 0.19, the flapping motion of symmetric up- and downstroke pitching (*α*_d_ = −15°, *α*_u_ = 15°) leads to the optimal propulsive efficiency *η*_p_ = 0.37 at the cost of zero mean lift coefficient (figure 5*b*) and efficiency *P_f_* = 0. When the flapping motion becomes asymmetric with *α*_u_ = 30° ∼ 45°, figure 5*b* shows that the ${\bar{C}}_{l}$, ${\bar{C}}_{m}$ and also *P_f_* increased dramatically, but the *η*_p_ is reduced. The complementary nature of *η*_p_ and *P_f_* can also be seen from figure 5*a*,*b* that the maximum *P_f_* always occur at small *St* with negative ${\bar{C}}_{m}$, where the FWR wing produces net drag instead of thrust.

Previous studies have shown that the propulsive efficiency of flapping aerofoils is closely related to the evolution of the flow structure on the wing. Triantafyllou *et al.* [6,7] studied the propulsive efficiency of 2D oscillating aerofoil and proposed that the optimal efficiency is obtained when an aerofoil is flapped at the frequency that results in the maximum amplification of the shed vortices, and the velocity profile behind the aerofoil is in the form of an inverted von Kármán vortex street indicative of a jet. Later, by using an inviscid panel method to investigate the wake structure of a 2D oscillating aerofoil, Jones *et al.* [26] noted a remarkable similarity between the simulated wake and the experiment wake structure, indicating that the formation of the well-defined wake structure is essentially an inviscid phenomenon.

At low *Re* and large AoA, the well-defined structure of the wake is complicated by the flow separation at the leading edge and interactions with the vortices shed from the trailing edge. Wang [25] studied the flow over an impulsively started 2D aerofoil using CFD method and observed that the thrust production is correlated with the time scale that governs the shedding of the LEV. He proposed that optimal efficiency is obtained when the duration of the flapping stroke is inside the ‘thrust window’ that exists before the LEV is shed.

**Figure 6.**
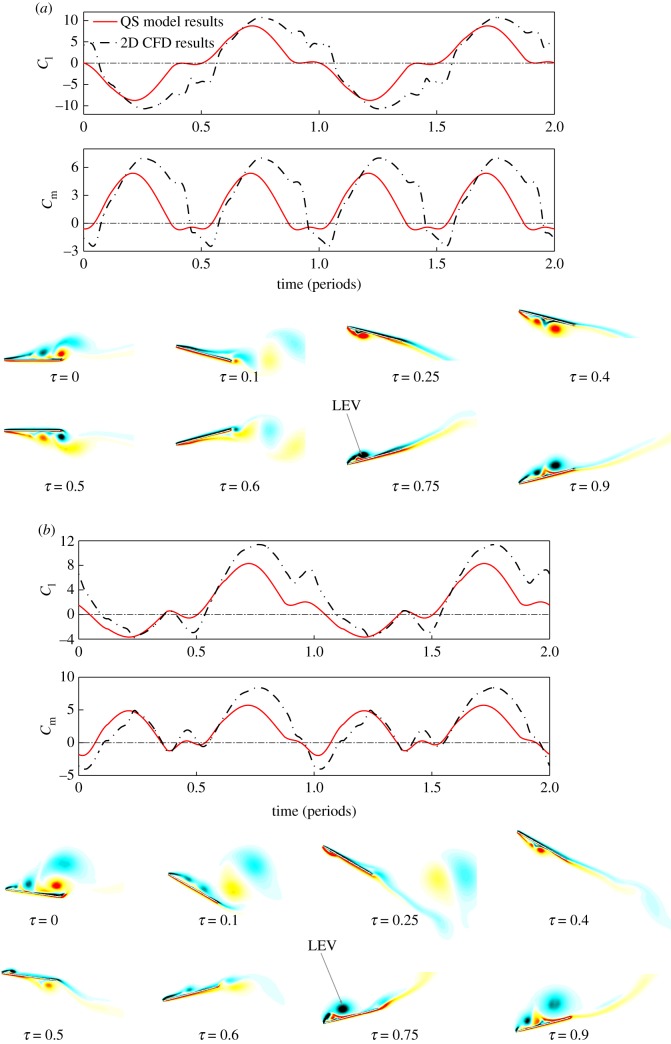
Time courses of *C*_l_ and *C*_m_ and vorticity contours for maximum *η*_p_ cases. (*a*) Symmetric case: *α*_u_ = 15°, *α*_d_ = −15° at *St* = 0.19; (*b*) asymmetric case: *α*_u_ = 30°, *α*_d_ = −15° at *S*t = 0.29.

In the view of the previous results in 2D, we have taken the optimal kinematic cases (shown in figure 5 and table 1) that result in the highest propulsive efficiency and efficiency of lift to investigate the 2D flow and forces. The 2D calculations are conducted for the cases with symmetric pitch angles: *α*_u_ = 15°, *α*_d_ = 15° at *St* = 0.19, and asymmetric pitch angles: *α*_u_ = 30°, *α*_d_ = −15° at *St* = 0.29 that yield the maximum *η*_p_; and the case with: *α*_u_ = 45°, *α*_d_ = −15° at *St* = 0.22 that yields the maximum *P_f_*. The calculated time courses of forces and flow for maximum *η*_p_ cases are shown in figure 6.

In figure 6, large thrust is produced in both the upstroke and downstroke for the two cases. In the symmetric pitching case, the production of thrust is equal in the up- and downstroke; while in the asymmetric pitching case, the thrust produced in the downstroke is larger than in the upstroke. Figure 6*a* shows that the LEVs of the symmetric pitching case are of equal strength on both sides of the wing in the up- and downstroke, which contributes equally large thrust in each stroke. By contrast, figure 6*b* shows that asymmetric LEVs are formed on the wing for the asymmetric pitching case. In the downstroke, a strong LEV is formed on the upper wing surface which contributes significant lift and thrust; while in the upstroke, a weak LEV is formed on the lower wing surface which contributes small thrust and negative lift.

It is observed that the scales of the LEVs associated with the above cases are relatively small. This is due to the small effective AoA at the given *St*. In general, the formation of LEV decreases the propulsive efficiency, as indicated in previous studies for oscillating 2D aerofoils [27–29]. However, LEVs also serve as a source of thrust production. It is noted that the *St* serves to balance these two effects. As shown in figure 5*b*, when *St* decreases from moderate to small value, a transition from large rotational moment to negative is observed, indicating a decrease of the effective AoA from large value to negative. Maximum propulsive efficiencies occur at medium *St* where small positive effective AoA forms small LEV, as shown in figure 6.

**Figure 7.**
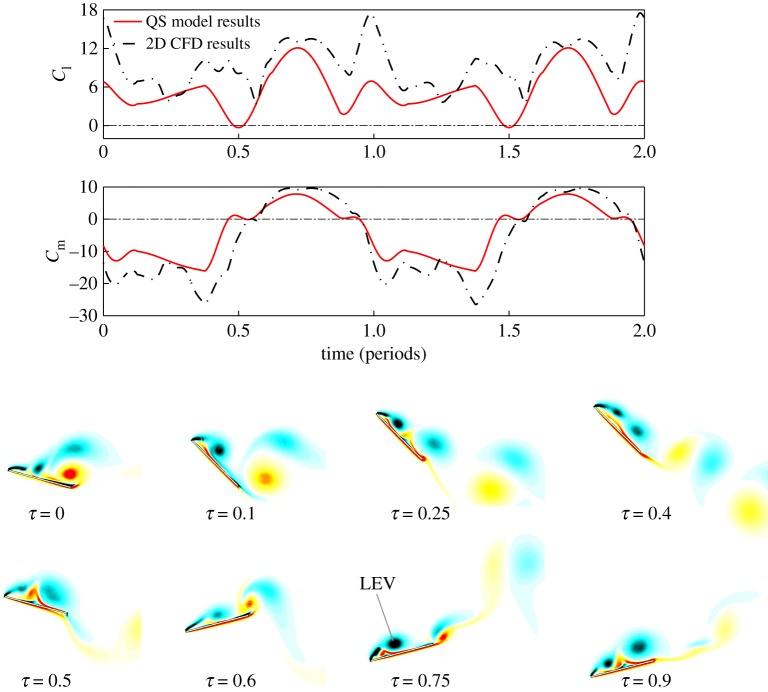
Time courses of *C*_l_ and *C*_m_ and vorticity contours for maximum *P_f_* case: *α*_u_ = 45°, *α*_d_ = −15° at *St* = 0.22.

Figure 7 shows the time courses of lift, rotational moment and 2D flow structure of the wing for the maximum *P_f_* case with an even larger pitch angle (*α*_u_ = 45°). The forces and moments of the 2D CFD results follow qualitatively the trends of the 3D QS forces and moments. However, disturbances of the 2D forces are observed due to the shedding of LEVs. In this case, lift is produced in both upstroke and downstroke. Large anti-rotating moment is produced in the upstroke and only a small rotational moment is produced in the downstroke. It is noted that in both up- and downstroke, large LEVs are formed on the upper surface of the wing and significant vortices shedding are observed near the end of each stroke. This is most likely due to the fact that the wing has consistently large effective AoA in a whole flapping cycle at this *St*.

From the above analysis, it is noted that as the wing pitch angles change from symmetric to asymmetric in the up- and downstroke, a transition of the LEV structure from symmetric to asymmetric is observed. The asymmetry of the LEV structure on the wing surface results in net lift production. However, as the flow forms large LEV in the downstroke, higher power is required to overcome the vertical lift force, which leads to a diminished propulsive efficiency. The transition of the flow structure from symmetry to asymmetry thus indicates a transfer of flapping energy from propulsion to weight suspension.

### 4.2. Aerodynamic efficiency of passive rotating wing

When the FWR wing is free to rotate horizontally, the rotation speed will reach an equilibrium state when the mean rotational moment over a flapping cycle is zero. The rotational equilibrium state of the FWR kinematics has been proposed for practical design of MAVs. Several previous studies have shown that this kinematics may have certain benefits in terms of system simplicity and aerodynamic efficiency compared with other conventional type (fixed wing, rotary wing and insect-like flapping wing) when applied for MAV design [13,15,16]. Apart from practical applications, the passive rotating kinematics also has a notable similarity with the cruising flight of natural flyers, where the flapping wings produce both lift and thrust, and the cruise speed is determined by the equilibrium of the body drag and the flapping propulsive thrust.

The present investigation focuses on the rotational equilibrium state of the FWR wing. As the wing produces no net propelling moment at this state, the efficiency for producing lift *P_f_* is of particular interest. In this investigation, the aerodynamic efficiency at the equilibrium state is compared with the maximum aerodynamic efficiency, which is the highest aerodynamic efficiency that can be obtained by actively tuning the rotation speed (or equivalently the *St*, as shown in figure 5) of the wing. The efficiency for producing lift at the rotational equilibrium state is denoted by *P_fe_*, while the maximum efficiency for the given wing pitch angles (*α*_u_ and *α*_d_) is indicated by *P_fm_*.

**Figure 8.**
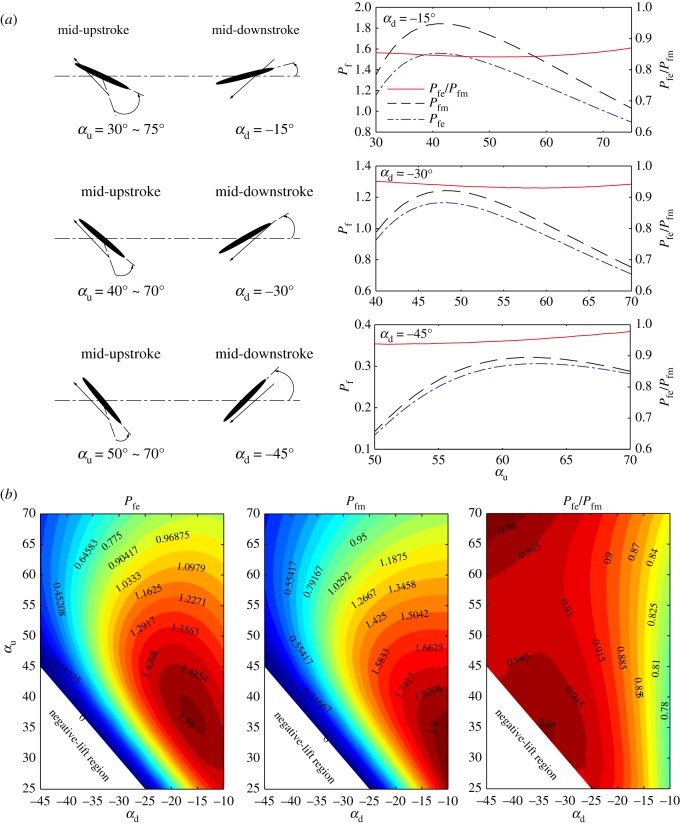
The variations of efficiency at equilibrium state *P_fe_*, the optimal efficiency *P_fm_* and the ratio $P_{fe}/P_{fm}$ with *α*_u_ and *α*_d_. (*a*) Variations of efficiencies with *α*_u_ for fixed *α*_d_ = −15°, −30° and −45°; (*b*) efficiency contours at $\alpha_{u}=25^{\circ }∼70^{\circ }$ and $\alpha_{d}=-45^{\circ }∼-10^{\circ }$.

In this investigation, the two efficiencies *P_fe_* and *P_fm_* are calculated with the downstroke wing pitch angle *α*_d_ prescribed between −45° and −10° and the upstroke wing pitch angle *α*_u_ prescribed between 25° and 70°. Specifically, the individual cases when *α*_d_ is fixed at −15°, −30° and −45° are taken out to analyse the efficiency variations with *α*_u_. For all the cases, the wing semi-span is taken as *R* = 0.098 m, wing aspect ratio λ = 3.6, the flapping frequency *f* = 10 Hz, flapping amplitude Φ = 70° and the *Re* is about 2500. The variation contours of *P_fe_*, *P_fm_* and the ratio *P_fe_*/*P_fm_* with *α*_u_ and *α*_d_ are shown in figure 8*b*. The efficiency curves with fixed *α*_d_ are shown in figure 8*a*. In figure 8*b*, the negative lift regions correspond to wing kinematic cases with larger negative *α*_d_ in the downstroke than positive *α*_u_ in the upstroke, which consequently yields negative mean lift force in a flapping cycle.

The results in figure 8 shows that, in general, the efficiencies (*P_fe_* and *P_fm_*) are sensitive to the variation of wing pitch angles (*α*_u_ and *α*_d_). Increasing the downstroke AoA *α*_d_ generally leads to decreases in the aerodynamic efficiencies; while the optimal upstroke AoA *α*_u_ for *P_fe_* and *P_fm_* always takes intermediate values between 30° and 60°, depending on *α*_d_. However, despite these variations with wing pitch angles, the efficiency at the equilibrium states *P_fe_* appears to be very close to the maximum efficiency *P_fm_*. In most of the investigated kinematic cases (i.e. *α*_d_ between −45° and −15°), the ratio $P_{fe}/P_{fm}$ is above 85%; furthermore, when the downstroke wing pitch angle is large (i.e. *α*_d_ between −45° and − 30°), the ratio $P_{fe}/P_{fm}$ reaches even higher value of above 90%.

The above results imply that for the passive rotating wing, the forces equilibrium of the flapping propulsive thrust and anti-rotating drag in the up- and downstrokes results in a wing kinematic state of high aerodynamic efficiency. It is therefore expected that the passive rotation kinematics may be favourable for bioinspired MAV design, because the rotation speed automatically converges to a high-efficiency state. Furthermore, because flapping wing flyers in cruising flight are in a state of equilibrium where the production of thrust by their flapping wings balances with the drag from the body, the above results imply that cruising flyers may also benefit from this natural equilibrium to gain high aerodynamic efficiency of lift production at this state.

In order to fully characterize the kinematics of the three statuses of the wing, i.e. the maximum propulsive efficiency state, the state with maximum efficiency of lift and the equilibrium state, the *St* of these respective states at different wing pitch angles are further calculated. It should be noted that because the *St* serves to determine the production of aerodynamic forces from propulsive thrust to anti-rotating drag, the *St* at equilibrium state stands in the middle of the other two states.

**Figure 9.**
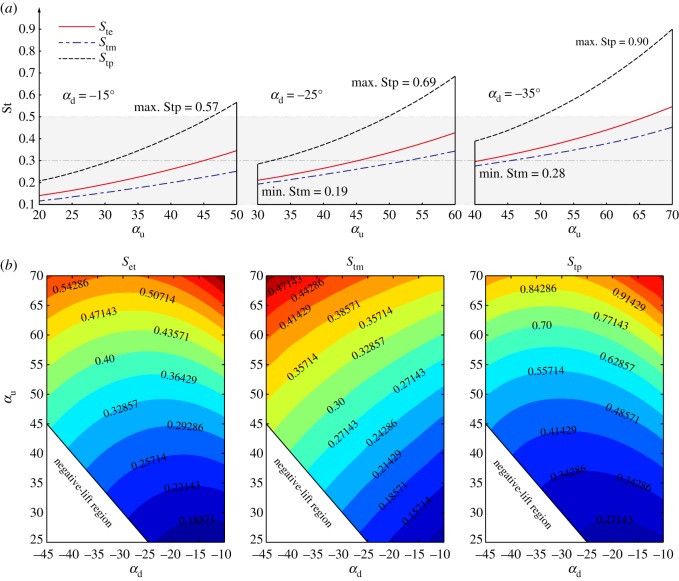
Distributions of *St* in typical states (*S_tp_*: maximum *η*_p_ state; *S_tm_*: maximum *P_f_* state and *S_te_*: rotational equilibrium state) with the wing pitch angles *α*_u_ and *α*_d_. (*a*) *S_te_*, *S_tm_* and *S_tp_* variations with *α*_u_ for fixed *α*_d_ = −15°, −25° and −35°; (*b*) contours of *S_te_*, *S_tm_* and *S_tp_* for $\alpha_{u}=25^{\circ }∼70^{\circ }$ and $\alpha_{d}=-45^{\circ }∼-10^{\circ }$.

Figure 9*a* shows the variations of *St* at the typical states with *α*_u_ for fixed *α*_d_ cases; the full contours of typical *St* for different wing pitch angles are shown in figure 9*b*. In figure 9, the *St* for maximum *η*_p_ states are denoted by *S_tp_*, for maximum *P_f_* states are denoted by *S_tm_*, and for equilibrium states are denoted by *S_te_*.

The variations in figure 9*a* show that the *St* for typical states (*S_te_*, *S_tm_* and *S_tp_*) increase monotonically with the increase of *α*_u_. Figure 9*a,b* shows that reducing the wing pitch angle (i.e. decrease *α*_u_ and increase *α*_d_) lowers the *St* for the high-efficiency states, indicating that higher efficiencies (*η*_p_ and *P_f_*) are obtained at higher rotation speed at smaller wing pitch angles (because *St* is inversely proportional to the rotation speed).

Figure 9*b* shows that for most of the kinematic cases with *α*_u_ between 25° and 70° and *α*_d_ between −45° and −10°, the *St* at equilibrium states *S_te_* and maximum *P_f_* states *S_tm_* fall in the interval between 0.1 and 0.5; while the *St* at maximum *η*_p_ states appears to be higher between 0.2 and 0.9 for most of the cases. Particularly, the distribution of the data shows that when the upstroke AoA is small, i.e. *α*_u_ less than 45°, nearly all the *St* for high-efficiency states *S_tm_*, *S_tp_* and the equilibrium state *S_te_* converge to the interval of 0.1 ∼ 0.5. The lower end of *St* (between 0.1 and 0.3) corresponds to higher *P_f_* states, while the higher end (between 0.2 and 0.5) corresponds to higher *η*_p_ states. The current results are in close agreement with previously reported data of flying animals in cruising flight, which show that many natural flyers cruise with *St* between 0.2 and 0.4 [8]. The above results imply that the various wing kinematics of flapping wing flyers in cruising flight may result in high aerodynamic efficiency states for both lift production and propulsion, although these two efficiencies cannot be optimized at the same time.

## 5. Conclusion

The aerodynamic efficiency of a novel FWR kinematics which combines vertical flapping motion and passive horizontal rotation is investigated by using a QS aerodynamic model and 2D CFD analysis. The propulsive efficiency *η*_p_ for producing horizontal thrust and the efficiency *P_f_* for producing vertical lift of the wing are investigated for a wing model of elliptical shape with wing semi-span *R* = 0.098 m, wing aspect ratio λ = 3.6, flapping vertically with a frequency of *f* = 10 Hz and amplitude Φ = 70° at the *Re* of 2500.

The calculated data show that both the propulsive efficiency *η*_p_ and efficiency of lift *P_f_* depend on the dimensionless *St* and wing pitch angles (*α*_u_ and *d*_u_). For small wing pitch angles (upstroke AoA *α*_u_ less than 45°), both efficiencies *η*_p_ and *P_f_* are found to peak at *St* between 0.1 and 0.5. However, these two efficiencies are complementary to each other: when maximum *η*_p_ is obtained, the *P_f_* is relatively low; while maximum *P_f_* always occurs when the flapping wing produces net drag instead of thrust. In particular, high efficiency of lift production is found when *St* is between 0.1 and 0.3, which is, in general, lower than the *St* for high propulsive efficiency (between 0.2 and 0.5). Further analyses of the 2D flow of the wing in typical kinematic states show that the production of lift and thrust is closely related to the LEV structure on the wing. Maximum *η*_p_ occurs when the wing forms small and symmetric LEVs in the up- and downstroke; while asymmetric LEVs large in the downstroke are associated with the production of lift and decrease in propulsive efficiency *η*_p_.

The rotational equilibrium state of the FWR kinematics is investigated. The results show that the aerodynamic efficiency at this state *P_fe_* is above 85% compared with the maximum efficiency *P_fm_* for a wide range of wing kinematics. Furthermore, systematic calculations show that most of the *St* for the high-efficiency states (maximum *P_f_* state and maximum *η*_p_ state) and the equilibrium state of the wing are within the interval of *St* between 0.1 and 0.5. These results show that the natural equilibrium between thrust and drag of the flapping wings result in a state of high aerodynamic efficiency. The agreement of our results with reported data of cruising flight of animals indicates that flapping wing flyers may benefit from this equilibrium to gain high efficiency for both lift and thrust production, although these two efficiencies cannot be optimized at the same time. The above results also have implications for bioinspired MAV design, because for the passive rotating kinematics of FWR, no fine tuning of the rotation speed is needed to achieve a high-efficiency state.

## Supplementary Material

Time courses of 2D forces and flow for different span-wise locations

## Data accessibility

All supporting data are made available either in the article or the electronic supplementary material.
