# Supplementary material for: Aerodynamic efficiency of a bioinspired flapping wing rotor at low Reynolds number
Source: R Soc Open Sci. 2018 Mar 14;5(3):171307. doi: 10.1098/rsos.171307 (PMC5882673; doi:10.1098/rsos.171307)
Supplement: Time courses of 2D forces and flow for different span-wise locations [file rsos171307supp1.docx]

Supplementary material for

‘Aerodynamic efficiency of a bio-inspired flapping wing rotor at low Reynolds number’ for *Royal Society Open Science*

H Li, S Guo (s.guo@cranfield.ac.uk)

| 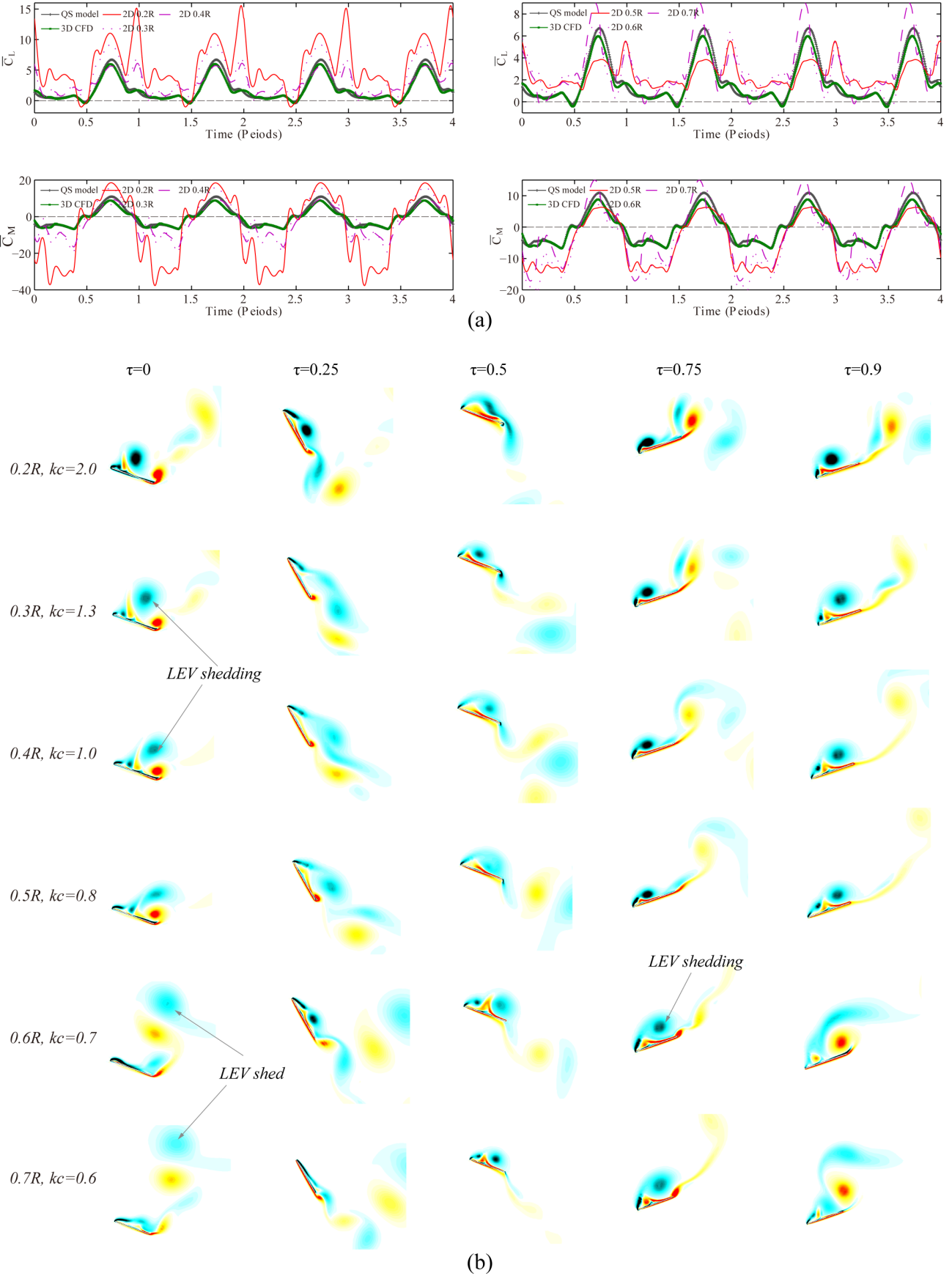 |
| --- |
| **Figure S1.** (a) Time courses of $C_{l}$ and $C_{m}$ by QS, 3D CFD and 2D CFD model at different chord-wise locations; (b) Contour of flow vorticity for 2D wing chords. *St*=0.45 for all cases; LEV tends to separate early as the chord location moves away from the wing root. |
